# Efficient techniques for genotype‐phenotype correlational analysis

**DOI:** 10.1186/1472-6947-13-41

**Published:** 2013-04-04

**Authors:** Subrata Saha, Sanguthevar Rajasekaran, Jinbo Bi, Sudipta Pathak

**Affiliations:** 1 Department of Computer Science and Engineering, University of Connecticut, Storrs, Connecticut, USA

**Keywords:** Feature Selection Algorithm (FSA), Gene Selection Algorithm (GSA), Multifactor Dimensionality Reduction (MDR), Random Projection (RP), Single‐Nucleotide Polymorphism (SNP), Support Vector Machine (SVM), Principal Component Analysis (PCA)

## Abstract

**Background:**

Single Nucleotide Polymorphisms (SNPs) are sequence variations found in individuals at some specific points in the genomic sequence. As SNPs are highly conserved throughout evolution and within a population, the map of SNPs serves as an excellent genotypic marker. Conventional SNPs analysis mechanisms suffer from large run times, inefficient memory usage, and frequent overestimation. In this paper, we propose efficient, scalable, and reliable algorithms to select a small subset of SNPs from a large set of SNPs which can together be employed to perform phenotypic classification.

**Methods:**

Our algorithms exploit the techniques of gene selection and random projections to identify a meaningful subset of SNPs. To the best of our knowledge, these techniques have not been employed before in the context of genotype‐phenotype correlations. Random projections are used to project the input data into a lower dimensional space (closely preserving distances). Gene selection is then applied on the projected data to identify a subset of the most relevant SNPs.

**Results:**

We have compared the performance of our algorithms with one of the currently known best algorithms called Multifactor Dimensionality Reduction (MDR), and Principal Component Analysis (PCA) technique. Experimental results demonstrate that our algorithms are superior in terms of accuracy as well as run time.

**Conclusions:**

In our proposed techniques, random projection is used to map data from a high dimensional space to a lower dimensional space, and thus overcomes the curse of dimensionality problem. From this space of reduced dimension, we select the best subset of attributes. It is a unique mechanism in the domain of SNPs analysis, and to the best of our knowledge it is not employed before. As revealed by our experimental results, our proposed techniques offer the potential of high accuracies while keeping the run times low.

## Background

A single‐nucleotide polymorphism (SNP) is defined as a DNA sequence variation where a single nucleotide, i.e., A, T, C, or G in the genomic sequence differs among the individuals of a biological species. It is the most common type of genetic variation among people. If CCGAATC and CCGAATA are two sequenced DNA fragments from two different individuals, these fragments differ in only one nucleotide position and this is called a SNP [1]. If we make comparisons between any two human genomic sequences side by side, they will be almost 99.9*%* identical [2]. Having 3.2 billion base‐pair genomes, individuals can have some 3.2 million differences in diploid genome. Most of the differences are due to SNPs. Even though most of the SNPs are of no biological significance or meaning, a fraction of the substitutions have functional consequence and these variations are the basis for the diversity found among humans [3]. SNPs are not evenly distributed across the whole genomic sequence. They occur more frequently in non‐coding regions than in coding regions of the genomic sequence. Most SNPs have no effect on health or development. Some of these genetic differences, however, have proven to be very important in the study of human health. Researchers have found SNPs that may help predict an individual’s response to certain drugs, susceptibility to environmental factors such as toxins, and risk of developing particular diseases. SNPs can also be used to track the inheritance of genes accused for disease within families. Future studies will work to identify SNPs associated with complex diseases such as heart disease, diabetes, and cancer.

In this paper, the main problem of interest is to take as input (say) two groups of individuals separated based on some phenotypes, together with their genotypes information and identify the most relevant SNPs that can explain the groupings. Our new approach is based on two paradigms: gene selection [4], and random projections [5] to identify a subset of SNPs from a set of SNPs that can altogether differentiate two groups of individuals efficiently and reliably within a short amount of time. In the first approach, we employ a feature selection algorithm (FSA) to identify the *k* most relevant SNPs (where *k* can be chosen by the user) to differentiate a group of individuals from another. To validate this approach, we computed the *p*‐value for each of the SNPs. It is found that a significant number of SNPs selected by the FSA has a very low *p*‐value. In the second approach, we employ random projections to project the original data into a space of dimension *d* (where *d* can be chosen by the user). We then compute a subset of dimensions which can together differentiate two groups of individuals. We have done this in two steps. We take the best *m* SNPs found by using the FSA. For each subject we keep only these *m* SNPs. The modified dataset is then projected onto a *k*‐dimensional space for various values of *k*. The FSA is then employed to identify a subset of dimensions that can best predict a particular class of subjects. Both of these approaches yield very good outcomes and our simulation results show that our proposed algorithms are indeed reliable, scalable, and efficient. They also outperform one of the currently best performing algorithms [6] in terms of accuracy and runtime.

The rest of this paper is organized as follows: Some background information and preliminaries are presented in the Background summary section. In this section, from among other things, we provide a brief introduction to *Support Vector Machine (SVM)*, and *Principal Component Analysis (PCA)*. In the Methods section we describe algorithms that we have employed in this study. Specifically, we discuss the *Feature Selection Algorithm* (FSA), *Random Projection* (RP), and *Multifactor Dimensionality Reduction* (MDR). Our Algorithms section describes the proposed algorithms. The performance of the algorithms is measured on real datasets and the results are presented in Results and discussions section. Conclusions section concludes the paper.

## Background summary

### Data source

In this paper, we have performed a candidate gene study for a complex human behavior disorder, drug dependency using scalable, and efficient computational techniques. Although candidate gene studies have their own inherent limitations (reviewed in [7]), the use of smaller focused arrays possibly represents a more practical approach for many studies than the use of large scale arrays such as genome wide association studies (GWAS). These focused arrays are able to overcome the issues of inadequate gene coverage by providing full coverage for a limited number of candidate genes. Such focused arrays offer the advantages of lower cost and lower false discovery rate, especially in situations where a dataset may have inadequate power due to size or other reasons. Our genetic markers were obtained in a study conducted by National Institute of Alcohol Abuse and Alcoholism (NIAAA). For details about our data readers are referred to [8]. According to [8], the panel SNPs that we use in our study are able to extract full haplotype information for candidate genes in alcoholism, other addictions and disorders of mood and anxiety.

### Feature selection

Feature selection techniques are used to efficiently select a subset of SNPs from a set of SNPs which can best define a system. They are different from other dimensionality reduction techniques like projection‐based (e.g., principal component analysis, random projections) or compression‐based (e.g., using information theory) techniques. The latter techniques do not alter the original representation of the variables but just select a subset of them to best describe a system. A comprehensive and detailed review on feature selection techniques in bioinformatics can be found in [9]. Machine learning techniques can also be applied in the domain of SNPs selection [10]. Support Vector Machine (SVM), Genetic Algorithm (GA), Simulated Annealing (SA), Principal Component Analysis (PCA), etc have been applied widely in bioinformatics. Examples of works that employ SVM are [11]‐[13]. [14] detects a subset of potential SNPs by using Simulated Annealing (SA) and also provides a comprehensive and detailed review of the current approaches to identify SNPs. PCA based research can be found, for example, in [15,16].

### Support Vector Machine (SVM)

Support Vector Machine (SVM) has been developed by Vapnik, et al. at AT&T Bell Laboratories [17,18] which is the basis of gene selection algorithm. Kernel‐based techniques (such as support vector machines, Bayes point machines, kernel principal component analysis, and Gaussian processes) represent a major development in machine learning algorithms. Support vector machines (SVMs) are a group of supervised learning methods that can be applied to classification or regression. They represent an extension to nonlinear models of the generalized portrait algorithm. The basic idea is to find a hyperplane which separates any given *d*‐dimensional data perfectly into two classes. Assume that we are given *l* training examples { *x*_*i*_,*y*_*i*_}, where each example has *d* inputs ($x_{i}\varepsilon {ℜ}^{d}$), and a class label *y*_*i*_*ε* {−1,1} where 1≤*i*≤*l*. Now, all the hyperplanes in ${ℜ}^{d}$ are parameterized by a vector (*w*), and a constant (*b*), expressed in the equation:

(1) w·x+b=0

Here *x* is a point on the hyperplane, *w* is a *n*‐dimensional vector perpendicular to the hyperplane, and *b* is the distance of the closest point on the hyperplane to the origin. Any such hyperplane (*w*,*b*) that separates the data leads to the function:

(2) f(x)=sign(w·x+b)

The hyperplane is found by solving the following problem:

Minimize $J=\frac{1}{2}∥w{∥}^{2}$; subject to *y*_*i*_(*w*·*x*_*i*_+*b*)−1≥0, where *i*=1,…,*l*.

To handle datasets that are not linearly separable, the notion of a “kernel induced feature space” has been introduced in the context of SVMs. The idea is to cast the data into a higher dimensional space where the data is separable. To do this, a mapping function *z*=*ϕ*(*x*) is defined that transforms the *d* dimensional input vector *x* into a (usually higher) *d*’ dimensional vector *z*. Whether the new training data {*ϕ*(*x*_*i*_),*y*_*i*_} is separable by a hyperplane depends on the choice of the mapping/kernel function. Some useful kernel functions are “polynomial kernel”, and “GAUSSIAN RBF kernel”. The *polynomial kernel* takes the form:

(3) $$K(x_{a},x_{b})={\left(x_{a}\cdot x_{b}+1\right)}^{p}$$

where *p* is a tunable parameter, which in practice varies from 1 to ∼ 10. Another popular one is the Gaussian RBF Kernel:

(4) $$K(x_{a},x_{b})=\;\exp\;\left(-\frac{∥x_{a}-x_{b}{∥}^{2}}{2\sigma^{2}}\right)$$

where *σ* is a tunable parameter. Using this kernel results in the classifier:

(5) $$f(x)=\text{sign}\left[\underset{i}{\sum }\alpha_{i}y_{i}\;\exp\;\left(-\frac{∥x-x_{i}{∥}^{2}}{2\sigma^{2}}\right)+b\right]$$

which is a Radial Basis Function, with the support vectors as the centers. More details and applications of SVM can be found in [19]‐[21].

### Principal Component Analysis (PCA)

Principal component analysis (PCA) is a technique that takes any high‐dimensional data to a lower‐dimensional form by using the dependencies among the variables, without losing too much information. PCA is one of the simplest and most robust ways of doing such dimensionality reduction. It employs orthogonal transformations to convert a set of observations of possibly correlated variables into a set of values of linearly uncorrelated variables. These uncorrelated variables are called principal components. PCA is also known as the Karhunen‐Loeve transformation, the Hotelling transformation, the method of empirical orthogonal functions, and singular value decomposition. The number of principal components is less than or equal to the number of original variables. Here the first principal component has the largest possible variance.

Assume that we are given *n*‐dimensional feature vectors and we want to summarize them by projecting it into a *d*‐dimensional subspace. The simplest solution is to find the projections which maximize the variance. The first principal component is the direction in the feature space along which the projections have the largest variance. The second principal component is the direction which maximizes the variance among all the directions orthogonal to the first. The *k*^*th*^ component is the variance‐maximizing direction orthogonal to the previous *k*−1 components. More information regarding PCA can be found in [22]‐[24].

## Methods

In this section we summarize the *Feature Selection Algorithm* as well as the technique of *Random Projections*. Feature selection is a classification algorithm based on SVMs. For any classification algorithm there will be two phases. In the first phase the classifier is trained with some training data and this phase can be thought of as a learning phase. In the second phase, the classifier’s accuracy is tested with test or treatment data. In this paper we utilize real data pertaining to subjects dependent on opium. We divide the set of input data into two groups: *G*_1_ contains all the non‐addicted subjects and *G*_2_ is the set of all addicted subjects. We train the classifier using a training set which consists of 50 percent data from each of *G*_1_ and *G*_2_ (randomly chosen), respectively. The test set is formed using the other 50 percent from *G*_1_ and *G*_2_, respectively.

### Feature selection

We have incorporated gene selection techniques [4] in our feature section algorithm to identify the correlation among the SNPs. The aim of gene selection algorithm is to identify the (smallest) subset of genes responsible for certain event(s). Please note that even though in the gene selection algorithm we refer to genes, the algorithm is generic and in general a ‘gene’ should be thought of as an arbitrary feature. Gene selection is based on SVMs and it takes as input *n* genes { *g*_1_,*g*_2_,*g*_3_,⋯,*g*_*n*_}, and *l* vectors { *v*_1_,*v*_2_,*v*_3_,⋯,*v*_*l*_}. As an example, each *v*_*i*_ could be an outcome of a microarray experiment and each vector could be of the following form: $v_{i}=\{x_{i}^{1},x_{i}^{2},x_{i}^{3},\cdots \;,x_{i}^{n},y_{i}\}$. Here $x_{i}^{j}$ is the expression level of the *j*^*th*^ gene *g*_*j*_ in experiment *i*. The value of *y*_*i*_ is either *+1* or *‐1* based on whether the event of interest is present in experiment *i* or not. The problem is to identify a subset of genes $\left\{g_{i}^{1},g_{i}^{2},g_{i}^{3},\cdots \;,g_{i}^{m}\right\}$ sufficient to predict the value of *y*_*i*_ in each experiment. Given a set of vectors, the gene selection algorithm learns to identify the minimum subset of genes needed to predict the event of interest and the prediction function. These vectors form the training set for the algorithm. Once trained, the algorithm is provided with a new set of data which is called the test set. The accuracy of gene selection is measured in the test set as a percentage of microarray data on which the algorithm correctly predicts the event of interest. The procedure solely relies on the concept of SVM.

Guyon, *et al.*[25] introduced a naive gene selection algorithm called sort‐SVM. Here the genes were sorted according to their corresponding weights and a subset of genes was selected from the sorted sequence and thus discarded the redundant information. The authors also developed an algorithm called Recursive Feature Elimination (RFE) which is based on the sensitivity analysis proposed by [26] where the change of cost function *DJ*(*i*) caused by removing a given feature *i* is approximately measured by expanding the cost function (*J*) in Taylor series to second order. As a result, genes can be selected based on the weight value of each feature. In each iteration train the SVM and obtain the weights for all the remaining genes and then eliminate the gene with the smallest weight until two genes are left. Following are the basic steps involved in the Recursive Feature Elimination (RFE) algorithm: (1) Train the linear SVM; (2) Compute weight for each gene; (3) Remove the gene with the smallest weight; and (4) Repeat steps 1, 2, and 3 until only 2 genes are left.

The gene selection algorithm of Song and Rajasekaran [4] is based on the ideas of combining the mutual information among the genes and incorporating correlation information to reject the redundant genes. The Greedy Correlation Incorporated Support Vector Machine (GCI‐SVM) algorithm of [4] can be briefly summarizes as follows: The SVM is trained only once and the genes are sorted according to the norm of the weight vectors corresponding to these genes. Then the sorted list of genes are examined starting from the second gene. The correlation of each of these genes with the first gene is computed until one whose correlation with the first one is less than a certain predefined threshold is found. At this stage this gene is moved to the second place. Now the genes starting from the third gene are examined and the correlation of each of these genes with the second gene is computed until a gene whose correlation with the second gene is less than the threshold is encountered. The above procedure is repeated until the end of the sorted genes is reached. In the last stage, genes based on this adjusted sorted genes are selected. GCI‐SVM brings the concept of sort‐SVM and RFE‐SVM altogether which makes it more efficient. These are: (1) GCI‐SVM incorporates correlation information to remove the redundant genes; (2) Sort‐SVM utilizes mutual information among genes but also may select redundant genes. GCI‐SVM uses RFE‐SVM concept which enables it to utilize the mutual information among genes; and (3) Other algorithms like RFE‐SVM make use of recursion to remove the redundant genes which is very time consuming. GCI‐SVM uses the combination of the above mentioned concepts together. This makes it time efficient. In a nutshell, GCI‐SVM works as follows: 

1. Compute the correlation coefficient for each pair of genes.

2. Train the SVM using the training data set.

3. Sort the genes based on their weight values.

4. Go through the sorted genes; pick those genes whose correlation with the previously picked genes is less than a threshold.

5. Move in order all picked genes to the front of the sequence; correspondingly, unpicked genes are moved to the end.

### Random projections

Mapping a set of points from a higher dimensional space to a lower dimensional space in such a way that the pair‐wise distances are closely preserved is a problem that has been studied widely. A finite set of *n* points in a *d*‐dimensional Euclidean space *R*^*d*^ can be represented by a matrix [*A*]_*n*×*d*_, where each row represents a point in *d* dimensions. The objective is to identify a mapping *f*:*R*^*d*^→*R*^*k*^ with negligible distortion in the distance between any pair of points. Here *k* is the dimension of the reduced space. Johnson and Lidenstrauss [27] have given an elegant randomized mapping such that the original pairwise distances are *ε*‐preserved in the *k*‐dimensional space.

#### Lemma

(*Johnson* &*Lindenstrauss*): Given *ε*> 0 and an integer *n*, let *k* be a positive integer such that *k*>*k*_0_=*O*(*ε*^−2^ log*n*). For every set *P* of *n* points in *R*^*d*^ there exists *f*:*R*^*d*^→*R*^*k*^ such that for all *u*, *v* in *P*:

(6) $$\;(1-\varepsilon )∥u-v{∥}^{2}\leq ∥f(u)-f(v){∥}^{2}\leq (1+\varepsilon )∥u-v{∥}^{2}$$

We can accomplish this mapping using the Achlioptas [5] method.

#### Theorem

Let *P* be an arbitrary set of *n* points in *R*^*d*^, represented by a *n*×*d* matrix *A*. Given *ε* and *β*>0, let,

(7) $$k_{0}=\frac{(4+2\beta )\;\log\;n}{\left(\frac{\varepsilon^{2}}{2}-\frac{\varepsilon^{3}}{3}\right)}.$$

For any integer *k* >*k*_0_, let *R* be a *d*×*k* random matrix with *R*(*i*,*j*) = *r*_*ij*_, where { *r*_*ij*_} are independent random variables from either one of the following probability distributions:

$$r_{\text{ij}}=\left\{\begin{array}{c} +1\;\text{with probability}\;\frac{1}{2}\\ -1\;\text{with probability}\;\frac{1}{2} \end{array}\right)$$

or,

$$r_{\text{ij}}=\sqrt{3}\left\{\begin{array}{c} +1\;\text{with probability}\;\frac{1}{6}\\ \;0\;\text{with probability}\;\frac{2}{3}\\ -1\;\text{with probability}\;\frac{1}{6}. \end{array}\right)$$

Let $E=\frac{1}{\sqrt{k}}\text{AR}$ and let *f*:*R*^*d*^→*R*^*k*^ map the *i*^*th*^ row of *A* to the *i*^*th*^ row of *E*. With a probability of at least 1−*n*^*β*^, for all *u*, *v* in *P*, the following inequality holds:

(8) $$(1-\varepsilon )∥u-v{∥}^{2}\leq ∥f(u)-f(v){∥}^{2}\leq (1+\varepsilon )∥u-v{∥}^{2}$$

Using one of the probability distributions we can construct [*R*]_*d*×*k*_. Multiplication of [*A*]_*n*×*d*_ and [*R*]_*d*×*k*_ maps *R*^*d*^ to *R*^*k*^.

### Multifactor Dimensionality Reduction (MDR)

Multifactor dimensionality reduction (MDR) is a data mining procedure which detects and characterizes combinations of attributes or independent variables that can altogether interact to influence a dependent or class variable. MDR is designed primarily to identify interactions among discrete variables that can together act as a binary classifier. It is considered as a nonparametric alternative to traditional statistical methods e.g., logistic regression. We can think of MDR as a constructive induction algorithm that can convert two or more discrete variables or attributes to a single variable or attribute. The method to create a new attribute or variable changes the representation space of the original data. The details of the MDR algorithm can be found in [6,28,29]. Authors in [30] develop the MDR‐PDT algorithm by merging the MDR method with the genotype‐Pedigree Disequilibrium Test (geno‐PDT). Unlike ordinary MDR, it can identify single‐locus effects or joint effects of multiple loci in families of diverse structure.

In the MDR algorithm, the observed data is divided into ten equal parts and a model is fit to each nine‐tenths of the data (the training data), and the remaining one‐tenth (the test data) is used to assess the accuracy to fit a model, thus using ten‐fold cross‐validation. Within each nine‐tenths of the data, a set of *n* factors is selected and their possible multifactor classes or cells are represented in *n* dimensional space. The steps of the MDR algorithm, according to [6], can be described as follows: 

1. In step one, the dataset is divided into multiple partitions to carry out cross‐validation. MDR can be performed without performing cross‐validation. But this is very infrequently done due to the potential for over‐fitting [31]. It tries to fit the data, learn a concept, build a model based on the learned concept and apply the concept to predict from unseen data.

2. A subset of *n* discrete variables or factors is selected from the set of all variables or factors.

3. The chosen *n* variables and their possible multifactor classes are organized into *n*‐dimensional space. For example, for two loci with three genotypes each, there are nine possible two locus‐genotype combinations. Then, the ratio of the number of cases to the number of controls is calculated within each multifactor class.

4. A reduction procedure on the *n* dimensional model to a one‐dimensional model is carried out. This is done by labeling each multifactor class in *n*‐dimensional space either as high‐risk or low risk. If the cases to controls ratio meets or exceeds some threshold (e.g., ≥1.0), it is called high‐risk. On the contrary, it is called low‐risk, if that threshold is not exceeded. By following the procedure stated above, a model for both cases and controls is formed by pooling high‐risk cells into one group and low‐risk cells into another group. This reduces the *n*‐dimensional model to a one‐dimensional model (i.e., having one variable with two multifactor classes – high risk and low risk). In a nutshell, among all of the two‐factor combinations, a single model that has the fewest misclassified individuals is selected.

5. The prediction error of each model is estimated by 10‐fold cross‐validation.

### Normalization

Normalization is the process of scaling any data so that it falls within a specified range. There are many methods of normalization, such as min‐max normalization, *z*‐score normalization, normalization by decimal scaling, etc.

#### Min‐max normalization

In min‐max normalization, a linear transformation is performed on the original data. Assume that the minimum and maximum values of an attribute *a* are given by *min*_*a*_, and *max*_*a*_. Min‐max normalization maps a value *v* to *v*’ in the new range [$\text{ne}w_{\text{mi}n_{a}}$, $\text{ne}w_{\text{ma}x_{a}}$] by computing:

(9) $$v^{\prime }=\frac{v-\text{mi}n_{a}}{\text{ma}x_{a}-\text{mi}n_{a}}(\text{ne}w_{\text{ma}x_{a}}-\text{ne}w_{\text{mi}n_{a}})+\text{ne}w_{\text{mi}n_{a}}$$

### Discretization

Discreetization is the method of placing continuous values into discrete buckets. The simplest method for discretization is to determine the minimum and maximum values of the attributes and then divide the range into user defined number of intervals of equal length. Each interval *I* is associated with an integer value *V*(*I*). Any value that falls in a particular interval *I* is mapped to the corresponding value *V*(*I*).

## Our algorithms

We have employed a dataset consisting of 1036 subjects denoted as *s*_1_, *s*_2_, *s*_3_, ⋯, *s*_1036_ and 1212 SNPs denoted as *snp*_1_, *snp*_2_, *snp*_3_, ⋯, *snp*_1212_. The subjects are divided into two major groups as described above. *Group*_1_ consists of subjects who are not addicted to opium and *Group*_2_ consists of subjects who are addicted to opium. The input dataset can be represented as a 1036×1212 matrix. Our goal is to identify a subset of SNPs that can correlate well with the grouping. We have employed several versions of our algorithms and the details are summarized below:

### Algorithm 1

In this algorithm [Please see Algorithm ??], we have used the feature selection algorithm to identify some of the best SNPs that can together identify two groups. The feature selection algorithm has two phases. In the first phase, the algorithm is trained with a training dataset. In this phase, the algorithm comes up with a model of concept. In the second phase of the algorithm, a test dataset is presented. The model learned in the first phase is used to classify the elements in the test dataset. As a result, the accuracy of the model learned can be computed. We divide the set of input data into two groups: *Group*_1_ contains all the non‐addicted subjects and *Group*_2_ is the set of all addicted subjects. We train the classifier using a training set which consists of 50 percent of data from each of *Group*_1_ and *Group*_2_ (data is chosen randomly), respectively. The test set is formed using the other 50 percent from *Group*_1_ and *Group*_2_, respectively. Details are given in Algorithm 1. FSA is trained with the training set and it builds a model of concept by using SVM. We have used a number of kernel methods in SVM including Linear, Polynomial, GAUSSIAN RBF, and Sigmoid to build the model. The result is a *n*×*m* matrix, where *n* is the number of subjects and *m* is most influential features (here SNPs) of the training dataset by which we can infer whether a particular subject of interest is in *Group*_1_ or *Group*_2_ with certain confidence (here accuracy). After finding such features we calculate *p*‐values of each feature and output it in increasing order of *p*‐values along with accuracy.

#### Algorithm 1 Finding best SNPs using FSA

### Algorithm 2

This algorithm [Please see Algorithm 2] employs random projections and feature selection algorithm together. The original dataset is trained with a training set to identify the best *m* SNPs. For each subject we keep only these SNPs. The modified dataset is projected onto *k*‐dimensions for various values of *k*. For each value of *k*, we compute accuracy using the feature selection algorithm. We have also employed Principal Component Analysis (PCA) instead of Random Projection (RP) in Algorithm 2. The result is very interesting and intuitive. It is described in the results section. Details of the algorithm are given in Algorithm 2. At first, the algorithm constructs training set and test set by choosing data randomly from *Group*_1_ and *Group*_2_. *Group*_1_ contains all the non‐addicted subjects and *Group*_2_ is the set of all addicted subjects. Training set consists of 50 percent of data from each of *Group*_1_ and *Group*_2_ (data is chosen randomly), respectively. The test set is formed using the other 50 percent from *Group*_1_ and *Group*_2_, respectively. FSA is then trained with the training set and it builds a model using linear SVM. The result is a *n*×*m* matrix where *n* is the number of subjects and *m* is most influential features. Through this set of features we can classify an unseen subject with certain accuracy. Random Projection (or PCA) is then applied onto these *m* features to reduce the feature space from *m* to *k*. Data normalization and data discretization are applied to this *n*×*k* matrix. The features and the accuracy are found with an invocation of Algorithm 1.

#### Algorithm 2 FSA with random projection

### Algorithm 3

In this algorithm [Please see ??], we compare the accuracy and runtime of our Feature Selection Algorithm (FSA) and Multifactor Dimensionality Reduction (MDR) Algorithm. The FSA has been trained with training dataset and the algorithm comes up with a model which is applied to the test dataset to identify the best possible combination of SNPs with the highest accuracy. The MDR takes the dataset as a combination of two classes and returns a model with one or more combination of SNPs, accuracy, and CV consistency. Details of the algorithm are described in Algorithm 3.

#### Algorithm 3 Comparison of FSA and MDR

## Results and discussions

We have done rigorous simulations to verify our proposed algorithms. These simulation results show that our algorithms indeed output significantly correct results which are illustrated next.

### Algorithm 1

At first, we compute the *p*‐values of each of the SNPs and sort them in increasing order of *p*‐values [Please see Table 1]. After that we identify the best 32 SNPs using the feature selection algorithm and validate these SNPs with the top SNPs found in the previous step based on *p*‐values. Here *p*‐value calculation is based on logistic regression based test, and each *p*‐value is calculated on a single SNP which is equivalent to a Chi‐square test. In our feature selection algorithm we have employed linear SVM as well as some well‐known kernels such as polynomial, GAUSSIAN RBF, and sigmoid to map the data from a space of low dimension to a space of high dimension [Please see Table 2, Table 3, Table 4, and Table 5].

**Table 1:** **SNPs based on *p*‐values**

**Rank**	**User defined SNP ID**	***p*‐value**
1	X192	6.161326E‐7
2	X592	3.907886E‐4
3	X114	4.902156E‐4
4	X483	6.466061E‐4
5	X569	9.02912E‐4
6	X253	0.001703205
7	X230	0.002096796
8	X1033	0.002481348
9	X275	0.00249018
10	X407	0.002598933

**Table 2:** Best 10 SNPs from the feature selection algorithm

**Rank**	**User defined SNP ID**	***p*‐value**
1	X114	4.902156E‐4
2	X458	0.002744632
3	X961	0.01519576
4	X704	0.01878017
5	X519	0.03505115
6	X100	0.0374225
7	X989	0.03831268
8	X216	0.04014865
9	X365	0.04285033
10	X1100	0.04807944

A subset of SNPs is selected by employing linear SVM in the Feature Selection Algorithm.

**Table 3:** Best 10 SNPs from the feature selection algorithm

**Rank**	**User defined SNP ID**	***p*‐value**
1	X114	4.902156E‐4
2	X458	0.002744632
3	X961	0.01519576
4	X704	0.01878017
5	X519	0.03505115
6	X100	0.0374225
7	X989	0.03831268
8	X216	0.04014865
9	X365	0.04285033
10	X1100	0.04807944

A subset of SNPs is selected by employing non‐linear SVM in the Feature Selection Algorithm. Here we have used Polynomial Kernel to map the set of SNPs from a low dimension to a high dimension.

**Table 4:** Best 10 SNPs from the feature selection algorithm

**Rank**	**User defined SNP ID**	***p*‐value**
1	X114	4.902156E‐4
2	X483	6.466061E‐4
3	X569	9.02912E‐4
4	X1033	0.002481348
5	X407	0.002598933
6	X1120	0.002646448
7	X709	0.002852061
8	X1200	0.003385855
9	X702	0.003590515
10	X178	0.00382676

A subset of SNPs is selected by employing non‐linear SVM in the Feature Selection Algorithm. Here we have used GAUSSIAN RBF Kernel to map the set of SNPs from a low dimension to a high dimension.

**Table 5:** Best 10 SNPs from the feature selection algorithm

**Rank**	**User defined SNP ID**	***p*‐value**
1	X114	4.902156E‐4
2	X483	6.466061E‐4
3	X569	9.02912E‐4
4	X1033	0.002481348
5	X407	0.002598933
6	X1120	0.002646448
7	X709	0.002852061
8	X1200	0.003385855
9	X702	0.003590515
10	X178	0.00382676

A subset of SNPs is selected by employing non‐linear SVM in the Feature Selection Algorithm. Here we have used Sigmoid Kernel to map the set of SNPs from a low dimension to a high dimension.

In the case of linear SVM, please note that the third best SNP (in terms of the *p*‐value) was one of the SNPs that the feature selection algorithm has picked [Please see Table 1, and Table 2]. A simple calculation shows that if we pick 32 SNPs at random, the probability that one of them will be one of the three best SNPs (in terms of *p*‐values) is 7.6%. This indicates that the feature selection algorithm is capable of identifying statistically significant SNPs. Also, the accuracy obtained is pretty good (73.805*%*) [Please see Table 6]. If we use the polynomial kernel by setting the parameter *p*=1 [Please see Equation 3], the same subset of SNPs is picked and the maximum accuracy is also identical as in the case of linear SVM [Please see Table 2, Table 3, and Table 6].

**Table 6:** Comparison of time and maximum accuracy of different methods

**Method name**	**Type**	**Maximum %** **accuracy**	**Execution** **time** **in minute**
FSA	Linear	73.805	5
FSA	Polynomial	73.805	0.17
FSA	GAUSSIAN RBF	45.698	0.15
FSA	Sigmoid	45.698	0.16
Random projection	FSA (Linear) + RP	73.805	–
PCA	FSA (Linear) + PCA	73.685	–
MDR	–	68.65	60

In this table “–” in the fourth column means much less time than for any other method.

In the case of GAUSSIAN RBF and sigmoid kernel, the best SNPs found by these kernels included five of the best SNPs picked by simple *p*‐value calculations [Please see Table 1, Table 4 and Table 5]. Here these kernels produce the same subset of SNPs and maximum accuracy [Please see Table 6]. Although by employing GAUSSIAN RBF and sigmoid the FSA is able to pick statistically significant genes compared to other methods described above, the accuracy obtained is very poor, i.e., 45.698% [Please see Table 6]. Please note that, we have chosen a large number of subsets of the SNPs and computed the quantities of interest for each such subset. The results are very similar.

### Algorithm 2

The second algorithm employs random projections and feature selection together. At first, we take the best 32 SNPs given by the feature selection algorithm and apply random projection over these dataset containing those SNPs and project the data onto a space of 5, 10, 15, 20, 25, and 30 dimensions. FSA is then applied to these reduced dimension to classify the subjects of interest. For all of the reduced dimensions, we always get the maximum accuracy of 73.805*%*. This result indeed indicates that according to the Achlioptas [5] method the mapping of a set of points from a higher dimensional space to a lower dimensional space closely preserves the pair‐wise distances. Without any loss of generality, we can thus project the large dataset into a lower dimensional space and can get the same result.

We have also employed PCA instead of random projection in Algorithm 2 to compare the accuracy given by our techniques. The procedure is the same as described above. After applying FSA we pick the top 32 SNPs and apply PCA technique to find principal components of the feature space. The result is a list containing the coefficients defining each component (sometimes referred to as loadings), the principal component scores, etc. We then compute the 1^*st*^ principal component scores to 15^*th*^ principal component scores of each of the SNPs for each subject. After this data normalization and data discretization have been applied. FSA is then applied to the reduced dimensions of 10, and 15 respectively to classify the subjects of interest. The resulted maximum accuracy found was 73.685*%* [Please see Table 6]. Clearly, our random projection method beats PCA in term of accuracy. Here again we see that random projections in conjunction with feature selection are very effective in identifying statistically significant features of the input.

### Algorithm 3

This approach validates the result of our feature selection algorithm that it indeed gives more accurate results than another well known algorithm called multifactor dimensionality reduction or MDR. MDR has been used to identify potential interacting loci in several phenotypes. MDR is a SVM‐like gene‐selection classifier algorithm. We have compared our gene selection algorithm with MDR in terms of accuracy and runtime. This comparison reveals that our algorithm outperforms MDR with respect to the time to calculate the best number of SNPs that can together serve as a classifier. We ran MDR with the time intervals of 10 minutes, 20 minutes, 30 minutes, and 60 minutes. The SNPs identified by our algorithms form the best subset of SNPs which are also given by MDR after running for 10 minutes and above whereas our FSA takes only 5 minutes to find the best SNPs with an accuracy of 73.805*%* [Please see Table 6] by employing linear SVM. But if we use polynomial kernel, FSA takes only 0.17 minutes [Please see Table 6]. Here accuracy is the measure of how much confident we can be that the resulting SNPs can together serve as a classifier to distinguish two groups of subjects. Both programs were run on the same 2.8 GHz dual core machine.

Java implementation of MDR has been used for the analysis of 1212 SNPs. There are three types of search methods available for driving the MDR, namely, exhaustive, forced and random. For each attribute count specified, *Exhaustive Method* exhaustively examines each combination of attributes. This search method has no options. *Forced Method* examines only one attribute combination. The combination must be specified in the provided text field as a comma‐separated list of attribute labels. The labels are case‐sensitive. And at last, for each attribute count specified, *Random Method* examines random combinations. There are two options here, namely, evaluations and runtime. *Evaluation Option* evaluates a given number of random combinations, for each attribute count specified. For each attribute count specified, *Runtime Option* evaluates random combinations for a given amount of time. As the *Exhaustive Method* runs indefinitely for the pair‐wise combination for the entire set of 1212 SNPs and the *Forced Method* is the totally irrelevant for our experiment, we used *Random Method* with the option of *Runtime*.

The best single‐locus model identified was *X*483, with a training and testing accuracy of 56.61*%* and 49.75*%*, respectively but the cross‐validation consistency was only 4 out of 10 after running for 5 minutes [Please see Table 7]. The best two‐locus model identified was *X*275, and *X*483, with a training and testing accuracy of 61.09*%* and 55.61*%*, respectively and cross‐validation consistency was 6 out of 10 [Please see Table 8]. After running for 15 minutes, MDR gave the best triple‐locus model consisting of *X*114, *X*216, and *X*1070 with a training and testing accuracy of 64.07*%* and 59.37*%*, respectively [Please see Table 9]. The cross‐validation consistency was 9 out of 10. On the contrary, our feature selection algorithm finds this combination after running for only 0.17 minutes with an accuracy of 73.085*%* without employing any randomness [Please see Table 6]. The ternary‐locus model identified after running for 30 minutes was *X*114, *X*315, *X*986, and X1039 with a training and testing accuracy of 68.51*%* and 52.42*%*, respectively. The cross‐validation consistency was 5 out of 10 [Please see Table 10]. After running for 60 minutes, MDR gave the best ternary‐locus model consisting of *X*114, *X*315, *X*986, and *X*1039 with a training and testing accuracy of 68.65*%*, and 53.20*%*, respectively. But the cross‐validation consistency was of only 4 out of 10 [Please see Table 11].

**Table 7:** MDR ‐ Time duration: 5 minutes

**Model**	**Training acc.**	**Testing acc.**	**CV cons.**
X483	0.5661	0.4975	4/10
X275 X483	0.6104	0.5688	7/10
X93 X275 X407	0.6314	0.5642	6/10
X228 X243 X665 X733	0.6806	0.5014	6/10

**Table 8:** MDR ‐ Time duration: 10 minutes

**Model**	**Training acc.**	**Testing acc.**	**CV cons.**
X483	0.5661	0.4975	4/10
X275 X483	0.6109	0.5561	6/10
X114 X216 X1070	0.6407	0.5937	9/10
X114 X315 X986 X1039	0.6842	0.5249	6/10

**Table 9:** MDR ‐ Time duration: 15 minutes

**Model**	**Training acc.**	**Testing acc.**	**CV cons.**
X483	0.5661	0.4975	4/10
X275 X483	0.6109	0.5561	6/10
X114 X216 X1070	0.6407	0.5937	9/10
X114 X315 X986 X1039	0.6844	0.5133	6/10

**Table 10:** MDR ‐ Time duration: 30 minutes

**Model**	**Training acc.**	**Testing acc.**	**CV cons.**
X483	0.5661	0.4975	4/10
X275 X483	0.6114	0.5555	5/10
X114 X216 X1070	0.6408	0.5810	8/10
X114 X315 X986 X1039	0.6851	0.5242	5/10

**Table 11:** MDR ‐ Time duration: 60 minutes

**Model**	**Training acc.**	**Testing acc.**	**CV cons.**
X483	0.5661	0.4975	4/10
X275 X702	0.6125	0.5534	5/10
X114 X216 X1070	0.6409	0.5781	8/10
X114 X315 X986 X1039	0.6865	0.5320	4/10

## Conclusions

A subset of single nucleotide polymorphisms (SNPs) can be used to capture the majority of the information of genotype‐phenotype association studies. The primary purpose of this research is to select a subset of SNPs while maximizing the power of detecting a significant association. From this point of view, we have proposed a number of approaches to find a subset of SNPs from the entire set to classify a set of individuals. Our proposed algorithms are indeed efficient, reliable, and scalable in terms of both accuracy and time complexity. Random projection has been used to project the data onto a lower dimensional space. A subset of attributes is then selected from this low dimensional space. To the best of our knowledge, random projection technique has not been employed before in the area of SNPs analysis. As revealed by our experimental results, these techniques offer the potential of high accuracies while keeping the run times low.

## Competing interests

All authors declare that they have no competing interests.

## Authors’ contributions

SS contributed to the implementation of the algorithms, testing and analysis, manuscript preparation, algorithms development, and performance analysis. SR contributed to algorithms development, analysis of the results, performance analysis, and manuscript preparation. JB contributed to data preparation, results analysis, and performance analysis. SP contributed to the implementation of the algorithms. All the authors have read and approved the final manuscript.

## Pre-publication history

The pre-publication history for this paper can be accessed here:

http://www.biomedcentral.com/1472-6947/13/41/prepub
